# Complete Genome Sequence of *Streptococcus agalactiae* Strain S25 Isolated from Peritoneal Liquid of Nile Tilapia

**DOI:** 10.1128/genomeA.00784-16

**Published:** 2016-08-04

**Authors:** Rafaella Menegheti Mainardi, Edson Antônio Lima Júnior, Jose Carlos Ribeiro Júnior, Vanerli Beloti, Anderson Oliveira Carmo, Evanguedes Kalapothakis, Daniela Dib Gonçalves, Santiago Benites Padua, Ulisses Pádua Pereira

**Affiliations:** a Department of Preventive Veterinary Medicine, Laboratory of Veterinary Bacteriology, Londrina State University, Londrina, Paraná, Brazil; b Department of Preventive Veterinary Medicine, Laboratory of Animal Products Inspection, Londrina State University, Londrina, Paraná, Brazil; c Department of General Biology, Institute of Biologic Sciences, Federal University of Minas Gerais, Belo Horizonte, Minas Gerais, Brazil; d Laboratory of Pharmacology and Toxicology of Natural Products, Universidade Paranaense, Umuarama, Paraná, Brazil; e Aquivet Aquatic Health Consulting Company, São José do Rio Preto, Brazil

## Abstract

*Streptococcus agalactiae* (Lancefield group B; GBS) is one of the major pathogens in fish production, especially in Nile tilapia (*Oreochromis niloticus*). The genomic characteristics of GBS isolated from fish must be more explored. Thus, we present here the genome of GBS S25, isolated from Nile tilapia from Brazil.

## GENOME ANNOUNCEMENT

*Streptococcus agalactiae* (group B *Streptococcus*, GBS) is a major pathogen to human, bovine, and many fish species (1, 2). Previous studies have shown, through molecular biology methods (1, 3–5) and genomic approaches (6–8), that GBS has a significant genetic diversity. However, few studies have been performed to characterize fish strains isolated from different outbreaks in recent years from distinct geographic regions.

*S. agalactiae* SA25 was isolated from a moribund fish with increased abdominal cavity due to excess fluid from a fish farm of Parana state, Brazil, in July 2015. The genome sequencing of *S. agalactiae* SA25 was performed using the Illumina MiSeq platform (Illumina Inc., San Diego, CA, USA) using a 300-bp paired-end library, which generated 2,291,552 reads. After sequencing, those reads were subjected to trimming and filtering using the CLC Genomics Workbench software (version 8.0.2), in which reads with an average Phred quality of less than 30 and with one or more ambiguities were removed. Finally, reads with a size less than 50 bp, as well as the last 10 nucleotides of the 3′ end of each read, were removed.

After the trimming, 2,003,478 reads were used in the assembly, which generated a genome coverage corresponding to ~360-fold, based on the reference genome size of 1,820,886 bp for *S. agalactiae* strain SA20-06 (NC_019048) (9). The genome of SA25 was assembled using CLC Genomics Workbench software. A total of 35 contigs were generated, with an *N*_50_ of 109,762 bp and largest and smallest contig sizes of 340,795 bp and 511 bp, respectively. These contigs were ordered using CONTIGuator software (10) against many genomes of the same species. The genome of *S. agalactiae* strain SA20-06 was picked as a reference due to better synteny and number of contigs mapped. The gaps were removed with recursive rounds of short reads mapped against the scaffold (11). The annotation step was performed using the NCBI Prokaryotic Genome Annotation Pipeline.

Finally, the complete genome of *S. agalactiae* strain SA25 (with no gaps) was completed with a total size of 1,838,989 nucleotides and with 1,880 putative open reading frames, 35.52% G+C content, seven rRNA operons, 76 tRNA genes, and 140 pseudogenes. The S25 genome harbors the main virulence factors described for the species. SA25 belongs to serotype Ib, and the genetic profile resulting from multilocus sequence typing of this strain is related to host adaptability for heterothermic hosts (clonal complex 552 and sequence type 552). Interestingly, this strain has shown little virulence in experimental infection assays in Nile tilapia, with an LD_50_ value greater than 10^5^ CFU/fish (unpublished data).

New studies of *S. agalactiae* strains isolated from fish are underway with the objective to find genomic differences that may justify the distinct profile of virulence and antibiotic resistance observed in the our laboratory routine.

### Nucleotide sequence accession numbers. 

The *Streptococcus agalactiae* SA25 genome sequence and annotation data have been deposited at DDBJ/EMBL/GenBank under the accession number CP015976. The version described in this paper is the first version, CP015976.1.
